# Impact of dose calculation algorithm on magnetic resonance imaging-only radiotherapy for prostate and glioma^☆^

**DOI:** 10.1016/j.phro.2026.101045

**Published:** 2026-07-20

**Authors:** Sacha af Wetterstedt, Emilia Persson, Christian Jamtheim Gustafsson, Adalsteinn Gunnlaugsson, Minna Lerner, Lars E. Olsson, Tobias Pommer

**Affiliations:** a Radiation Physics, Department of Hematology, Oncology, and Radiation Physics, Skåne University Hospital, Lund, Sweden; b Department of Translational Medicine, Medical Radiation Physics, Lund University, Malmö, Sweden; c Department of Hematology, Oncology, and Radiation Physics, Skåne University Hospital, Lund, Sweden

**Keywords:** MRI-only radiotherapy, Synthetic CT, Acuros XB, Dose calculation algorithm, Prostate cancer, Glioma

## Abstract

**Background and Purpose:**

Synthetic computed tomography (sCT) enables magnetic resonance imaging (MRI)-only radiotherapy by providing electron density information for dose calculation. While sCT-based planning has been proven sufficiently accurate for convolution-based dose calculation algorithms, linear Boltzmann transport equation (LBTE)-based approaches exhibit higher sensitivity to tissue heterogeneities. This study evaluated the accuracy of both dose calculation algorithm types for MRI-only in prostate and glioma radiotherapy.

**Materials and methods:**

Clinical treatment plans for thirty-nine prostate cancer patients and seventeen glioma patients were recalculated on atlas- and deep learning (DL)-based sCTs and conventional CT using convolution- and LBTE-based algorithms. Target dose metrics were analyzed as relative differences, while organs of interest (OOI) dose criteria were evaluated as absolute differences between sCT- and CT-based calculations. Statistical significance was assessed using paired *t*-tests (α = 0.05).

**Results:**

For target volumes, statistically significant differences between sCT- and CT-based calculations were observed for both dose calculation algorithms (*p* < 0.001). In prostate patients, mean differences in target dose metrics were ≤ 1.4% for the LBTE-based algorithm and ≤ 0.5% for the convolution-based algorithm. Slightly smaller dose differences were observed for glioma patients, ≤ 0.8% and ≤ 0.5%, respectively. OOI dose differences were small (≤ 0.6%). The LBTE-based algorithm consistently yielded the largest dose differences between CT and sCT.

**Conclusions:**

Although the LBTE-based approach demonstrated higher sensitivity to sCT-related CT number discrepancies than the convolution-based dose calculation algorithm, the resulting dose differences remained within clinically acceptable limits, supporting the robustness of MRI-only radiotherapy for both atlas- and DL-based sCTs.

## Highlights

• Dose calculation algorithm influenced synthetic image dose differences.
• Linear Boltzmann dose calculation yielded twofold larger dose differences.
• Planning target volume dose differences remained ≤1% for prostate and glioma.
• Dose differences remained within clinically acceptable limits.

## Graphical abstract

**Figure:**
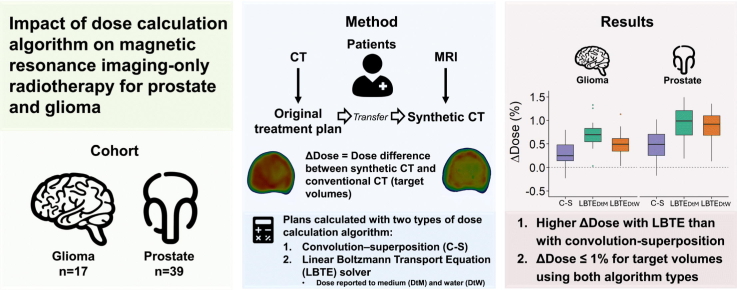

## 1 Introduction

In radiotherapy, a combination of image modalities is often utilized in order to obtain an appropriate basis for accurate target and organ of interest (OOI) delineation as well as providing the electron density information required for dose calculation [1]. In the brain and pelvic regions, this is commonly achieved using a combination of magnetic resonance imaging (MRI) and computed tomography (CT) [2].

Synthetic CT (sCT) images created directly from MRI reduce the total geometric uncertainties in the radiotherapy process by eliminating the registration step between different image modalities [3], [4]. In addition, since sCTs provide the CT number information required for electron density conversion and dose calculation in the treatment planning system (TPS) [5], [6], the conventional CT can be removed from the patient treatment chain. This reduces radiation exposure and streamlines the clinical workflow with potential reductions in staffing and equipment costs [7], [8]. Commercially available software for generating sCTs from magnetic resonance (MR) images uses a variety of techniques, including atlas-based anatomical bulk density replacement and tissue-classification approaches [5], [6], voxel- or patch-based intensity mapping [9], and, more recently, deep learning (DL)-based algorithms [10].

For prostate and brain MRI-only treatments, the conversion to sCT images yields a CT number mapping sufficiently accurate for dose calculations using a pencil beam convolution-superposition algorithm, which utilizes multidirectional photon scatter kernels to account for anisotropic tissue heterogeneities [5], [9]. While computationally efficient and widely adopted in clinical practice, such algorithms exhibit reduced accuracy in regions with steep density gradients, such as intersections between tissue and air or bone [11].

In contrast, Linear Boltzmann Transport Equation (LBTE)-based dose calculation algorithms directly account for tissue heterogeneities by numerically solving the transport equation [12]. As a result, they generally display higher dose calculation sensitivity to the CT number variations at interfaces between tissues with differing electron density (e.g. bone, muscle, air), in comparison to convolution-superposition algorithms, and yield dose calculation results similar to gold standard Monte Carlo methods [13], [14], [15]. However, the performance of LBTE-based dose calculation algorithms in combination with sCT-based dose calculations for prostate and brain treatments has not been thoroughly investigated. Previous studies and reviews of MRI-only radiotherapy have either not specifically evaluated the impact of the dose calculation algorithm or have been limited to a single sCT generation method [16]. We hypothesize that CT number discrepancies between sCTs and their corresponding CTs result in larger dose differences when using an LBTE-based dose calculation algorithm compared to using a pencil beam convolution-superposition algorithm, implicating a need to assess the effect of algorithm selection.

The aim of this work was to evaluate the accuracy of an LBTE-based dose calculation algorithm for MRI-based treatment planning using atlas- and DL-based sCTs in prostate and brain radiotherapy.

## 2 Materials and methods

### 2.1 Patient inclusion

Thirty-nine prostate cancer patients, receiving radiotherapy at Skåne University Hospital, were included in this work. The patients were previously included in the MR-PROTECT study [17] and therefore had both MR images required for generating sCTs as well as conventional CT images (Lund ethical approval board DNR 2016/1033). The patients were prescribed radiotherapy to the prostate gland only, with a 7 mm margin and a total dose of 78 Gy in 39 fractions, in accordance with the clinical protocol present at the time of treatment.

Seventeen clinical glioma patients, receiving radiotherapy at Skåne University Hospital, were additionally included in this work. The patients constituted the cohort utilized for clinical implementation of the MRI-only workflow for glioma patients in 2023, where CT and sCT dose calculations were compared to each other (Lund ethical approval board DNR 2013/742). The majority of patients were prescribed 60 Gy in 30 fractions. The planning target volume (PTV) volumes ranged between 144.3 and 548.9 cm^3^ with varied locations within the cranium.

### 2.2 MRI and sCT

All prostate cancer patients underwent MRI on a GE Discovery 750 W 3.0 T (GE Healthcare, Chicago, Illinois, USA). The patients were scanned using a large field-of-view T2-weighted (T2w) sequence. The MR images were used for sCT generation, target and OOI delineation, and gold fiducial marker identification.

All glioma patients were scanned on a GE SIGNA Architect 3.0 T (GE Healthcare, Chicago, Illinois, USA). The scans included four different Dixon sequences; Water, Fat, InPhase and OutPhase.

At the time of clinical treatment for the prostate cancer patients (2017–2018), sCTs were created using the commercial software MriPlanner™ v.1.1.2 (Spectronic Medical AB, Helsingborg, Sweden). This software version utilized an automated atlas-based method to convert the T2w MR images to sCTs by identifying areas of certain tissue-classes in the MR images and assigning them with CT numbers pre-defined within the model [18].

For the purpose of this study, sCTs were also generated with MriPlanner v2.4.14 using the same T2w MR images. This newer version of the software uses a DL-based approach, where a spatially varying affine transfer function is used to convert the MRI into an sCT image. The transfer function coefficients are estimated using a deep convolutional neural network which has been trained on reference MR and CT data [18]. The same sCT software version was used for the glioma cohort.

### 2.3 Conventional CT and image registration

The conventional CTs serving as ground truth for the prostate and glioma sCTs were obtained using a Siemens Somatom Definition AS+ (Siemens Healthineers, Erlangen, Germany), 120 kVp tube voltage and a slice thickness of 3.0 mm (prostate) and 2.0 mm (glioma). The prostate CTs were resampled in Aria (Varian Medical Systems, Palo Alto, CA, USA) using interpolation to the same slice thickness as the corresponding prostate sCTs (2.5 mm) in order to align the dose matrices prior to dose evaluation. For the glioma cohort, both sCT and CT images already had the same slice thickness (2.0 mm). The sCTs were rigidly registered in Aria v18.0, using 3 degrees of freedom (x,y,z), to their corresponding CTs using an automatic bone anatomy match with a CT number range of 200–1700 HU.

The target and OOI structures, originally delineated in the clinical workflow by radiation oncologists on the T2w images, were then rigidly propagated to the new sCTs and resampled CTs. Due to the limited sCT image size, the copied couch contours were originally truncated and therefore reassigned, including automatic image expansion performed by the TPS, where voxels with a CT number of −1000 HU were added until the image size reached 57 × 57 cm^2^.

For prostate cancer patients, the sCT software automatically replaces any air inside the body contour with water-equivalent material with the purpose of removing the uncertainties introduced by keeping a volatile gas structure in the reference image material [18]. To account for this and avoid the influence of air within or near the treatment beam path on the calculated dose, any air in the same cranio-caudal level as the PTV with a 3 cm margin was manually delineated using an adaptive brush tool and visually verified before its density was set to water.

### 2.4 Treatment planning

All original sCT prostate and glioma plans were created within the clinical MRI-only workflow for Varian TrueBeam accelerators in the Eclipse TPS with volumetric modulated arc therapy (VMAT) technique.

A total of 39 prostate plans used either one arc (1 case) or two arcs (38 cases) and 10 MV energy, with a total number of monitor units (MUs) per Gy ranging between 284 and 364. OOIs for plan optimization included the rectum, bladder, femoral heads, genitalia, and in 37 out of 39 cases also the penile bulb. Additionally, a general normal-tissue objective was applied during optimization.

A total of 17 glioma cases were planned using 6 MV, and either two arcs (10 cases) or three arcs (7 cases). The arcs were arranged using either coplanar couch angles (7 cases) or a non-coplanar setup with a combination of a 0 degree couch angle arc complemented by a 45 and/or 85 degree couch angle for the other arc/arcs (10 cases). The total number of MUs per Gy ranged between 186 and 295. Depending on location, a variety of OOIs were used for optimization, typically including a subset of the chiasm, optic nerves, brainstem, eyes, cochlea, pituitary, lacrimal glands and hippocampus, along a general normal-tissue objective.

### 2.5 Dose calculation

The original clinical prostate treatment plans were copied to the new DL-based sCTs and to the resampled CTs. The glioma treatment plans, originally planned on the DL-based sCTs, were also copied and assigned to the corresponding conventional CTs.

The treatment plans were calculated using two separate algorithms: the Anisotropic Analytical Algorithm (AAA) v15.6.05, a 3D pencil beam convolution-superposition algorithm, and Acuros XB v15.6.05, an LBTE-based dose calculation algorithm. The dose grid resolution remained the same as in the clinical situation; 2.5 mm in all three directions for the prostate cases, and 1.0 × 1.0 × 2.0 mm^3^ for the glioma cases. Dose calculation on the new sCT and conventional CT was performed while maintaining the field setup from the clinical plan. For the Acuros XB calculations, the physical material table Acuros XB-13.5 was applied. Both the “Dose-to-water” and “Dose-to-medium” reporting modes were used. Hence, there was a total of six dose calculations for each patient: AAA, Acuros XB (dose-to-water) and Acuros XB (dose-to-medium), each calculated on both sCT and CT.

### 2.6 Dose distribution evaluation

Following dose calculation, an in-house script extracted dose volume histogram (DVH) data directly from the Aria database. The structure definitions and sampling across all image datasets and dose calculation algorithms was manually verified.

For target volumes, the DVH metrics used clinically at the institution were evaluated; for prostate patients PTV D_95%_ and clinical target volume (CTV) D_99%_, and for glioma patients PTV D_98%_ and gross tumour volume (GTV) D_98%_. OOIs were also evaluated using clinical criteria, such as mean dose and volume-based DVH metrics.

The dose differences were calculated as the difference between sCT-based and CT-based dose calculations for target volumes, relative to the corresponding CT-based dose metric, and as absolute dose or absolute volume differences for OOIs. All comparisons were performed separately for AAA and Acuros XB (both dose reporting modes), in order to assess algorithm-dependent sensitivity to CT number discrepancies between sCT and CT.

Statistical analysis was performed using paired *t*-tests to determine whether the mean dose difference between sCT- and CT-based calculations was statistically significant. Additional paired comparisons between dose calculation algorithms were performed on the dose differences between CT and sCT in order to assess algorithm-dependent sensitivity to CT number discrepancies. Normality of the paired dose differences was assessed using the Shapiro–Wilk test [19]. Minor deviations from normality were observed for some paired dose difference distributions in the prostate cohort for Acuros XB, but paired *t*-tests were considered appropriate due to their robustness and the sample size. Consistent results were obtained using non-parametric testing. A significance level of α = 0.05 was used for all tests.

## 3 Results

For target volumes, statistically significant differences were observed in both the prostate and glioma cohorts for both dose calculation algorithms (*p* < 0.001 for all target metrics; Table 1, Table 2). The LBTE-based Acuros XB algorithm demonstrated mean differences in clinically used PTV dose metrics ≤ 1.0% for prostate patients and ≤ 0.8% for glioma patients. For the convolution-based AAA algorithm, the corresponding differences were ≤ 0.5% for both cohorts. In the prostate cohort, the mean OOI dose differences in volume-based DVH metrics were ≤ 0.4 percentage points and ≤ 0.35 Gy in dose-based DVH metrics. In the glioma cohort, the mean OOI dose differences were ≤ 0.7 Gy for both dose calculation algorithms. In contrast to the prostate cohort, no systematic shift towards either negative or positive dose differences between sCT- and CT-based calculations was observed for the glioma OOIs. None of the observed differences were statistically significant (*p* > 0.05).

**Table 1:** Mean dose differences between sCT- and CT-based dose calculations for prostate patients (*n* = 39, prescribed dose 78 Gy/39 fr) along with ranges within brackets for clinically used dose metrics. Target volumes (PTV and CTV) are expressed as relative percentage differences, while organs-of-interest dose criteria are reported as absolute differences in Gy or percentage points (pp). L = left, R = right. Dose calculations were performed using Acuros XB (dose reporting: both dose-to-medium and dose-to-water) and AAA. The sCTs are DL-based. *P*-values were derived from paired *t*-tests.

Mean dose difference between DL-based sCT and CT – Prostate						
	AAA	p-value	Acuros XB, dose-to-medium	p-value	Acuros XB, dose-to-water	p-value
PTV D_95%_ (%)	0.5 (−0.3 to 1.0)	<0.001	1.0 (0.2 to 1.6)	<0.001	0.8 (0.1 to 1.4)	<0.001
PTV D_mean_ (%)	0.5 (−0.2 to 1.0)	<0.001	0.9 (0.2 to 1.5)	<0.001	0.9 (0.1 to 1.4)	<0.001
CTV D_99%_ (%)	0.5 (−0.1 to 1.1)	<0.001	1.4 (0.7 to 2.5)	<0.001	1.0 (−0.1 to 1.8)	<0.001
Rectum V_70Gy_ (pp)	0.2 (−0.6 to 0.7)	<0.001	0.3 (−0.5 to 0.9)	<0.001	0.4 (−0.3 to 1.0)	<0.001
Bladder V_65Gy_ (pp)	0.3 (−0.1 to 1.1)	<0.001	0.4 (−0.1 to 1.4)	<0.001	0.4 (−0.1 to 1.4)	<0.001
Bladder D_mean_ (Gy)	0.3 (−0.1 to 0.9)	<0.001	0.3 (−0.1 to 1.0)	<0.001	0.4 (−0.1 to 1.0)	<0.001
Femoral head L D_2%_ (Gy)	0.1 (−0.3 to 0.6)	<0.001	0.2 (−0.1 to 0.7)	<0.001	0.2 (−0.2 to 0.7)	<0.001
Femoral head R D_2%_ (Gy)	0.2 (−0.2 to 0.4)	<0.001	0.2 (−0.1 to 0.5)	0.001	0.1 (−0.2 to 0.6)	<0.001
Femoral head L D_mean_ (Gy)	0.1 (−0.3 to 0.4)	<0.001	0.2 (−0.2 to 0.5)	<0.001	0.1 (−0.3 to 0.4)	<0.001
Femoral head R D_mean_ (Gy)	0.1 (−0.1 to 0.4)	<0.001	0.2 (−0.1 to 0.4)	<0.001	0.1 (−0.2 to 0.5)	<0.001

**Table 2:** Mean dose differences between sCT- and CT-based dose calculations for glioma patients (*n* = 17, varying dose prescriptions) along with ranges within brackets for clinically used dose metrics. Target volumes (PTV and GTV) are expressed as relative percentage differences, while organs-of-interest dose criteria are reported as absolute differences in Gy. L = left, R = right. Dose calculations were performed using Acuros XB (dose reporting: both dose-to-medium and dose-to-water) and AAA. The sCTs are DL-based. P-values were derived from paired t-tests.

Mean dose difference between DL-based sCT and CT – Glioma						
	AAA	p-value	Acuros XB, dose-to-medium	p-value	Acuros XB, dose-to-water	p-value
PTV D_98%_ (%)	0.5 (0.0 to 1.5)	<0.001	0.8 (0.3 to 2.1)	<0.001	0.8 (0.0 to 2.4)	<0.001
PTV D_mean_ (%)	0.3 (−0.2 to 0.8)	<0.001	0.7 (0.0 to 1.3)	<0.001	0.5 (0.0 to 1.1)	<0.001
GTV D_98%_ (%)	0.3 (−0.2 to 0.8)	<0.001	0.7 (−0.1 to 1.4)	<0.001	0.5 (−0.1 to 1.0)	<0.001
Chiasm D_2%_ (Gy)	−0.3 (−4.7 to 2.0)	0.435	0.0 (−4.0 to 1.7)	0.895	−0.1 (−4.0 to 1.8)	0.435
Cochlea L D_mean_ (Gy)	−0.6 (−3.6 to 0.5)	0.136	−0.7 (−3.2 to 0.3)	0.090	−0.6 (−3.6 to 0.5)	0.136
Cochlea R D_mean_ (Gy)	0.4 (−0.3 to 3.6)	0.215	0.3 (−0.4 to 3.4)	0.317	0.4 (−0.3 to 3.6)	0.215
Pituitary D_mean_ (Gy)	0.1 (−0.7 to 1.6)	0.578	0.2 (−0.6 to 1.8)	0.402	0.1 (−0.7 to 1.6)	0.578
Lacrimal gland L D_mean_ (Gy)	0.0 (−0.5 to 1.1)	0.922	0.1 (−0.5 to 1.2)	0.877	0.0 (−0.5 to 1.1)	0.922
Lacrimal gland R D_mean_ (Gy)	0.0 (−0.1 to 0.1)	0.984	0.0 (−0.1 to 0.1)	0.416	0.0 (−0.1 to 0.1)	0.984
Hippocampus L D_mean_ (Gy)	−0.1 (−0.6 to 1.0)	0.647	0.0 (−0.7 to 1.2)	0.836	−0.1 (−0.6 to 1.0)	0.647
Hippocampus R D_mean_ (Gy)	0.0 (−0.4 to 0.6)	0.534	0.1 (−0.4 to 0.8)	0.165	0.0 (−0.4 to 0.6)	0.534

**Fig. 1:**
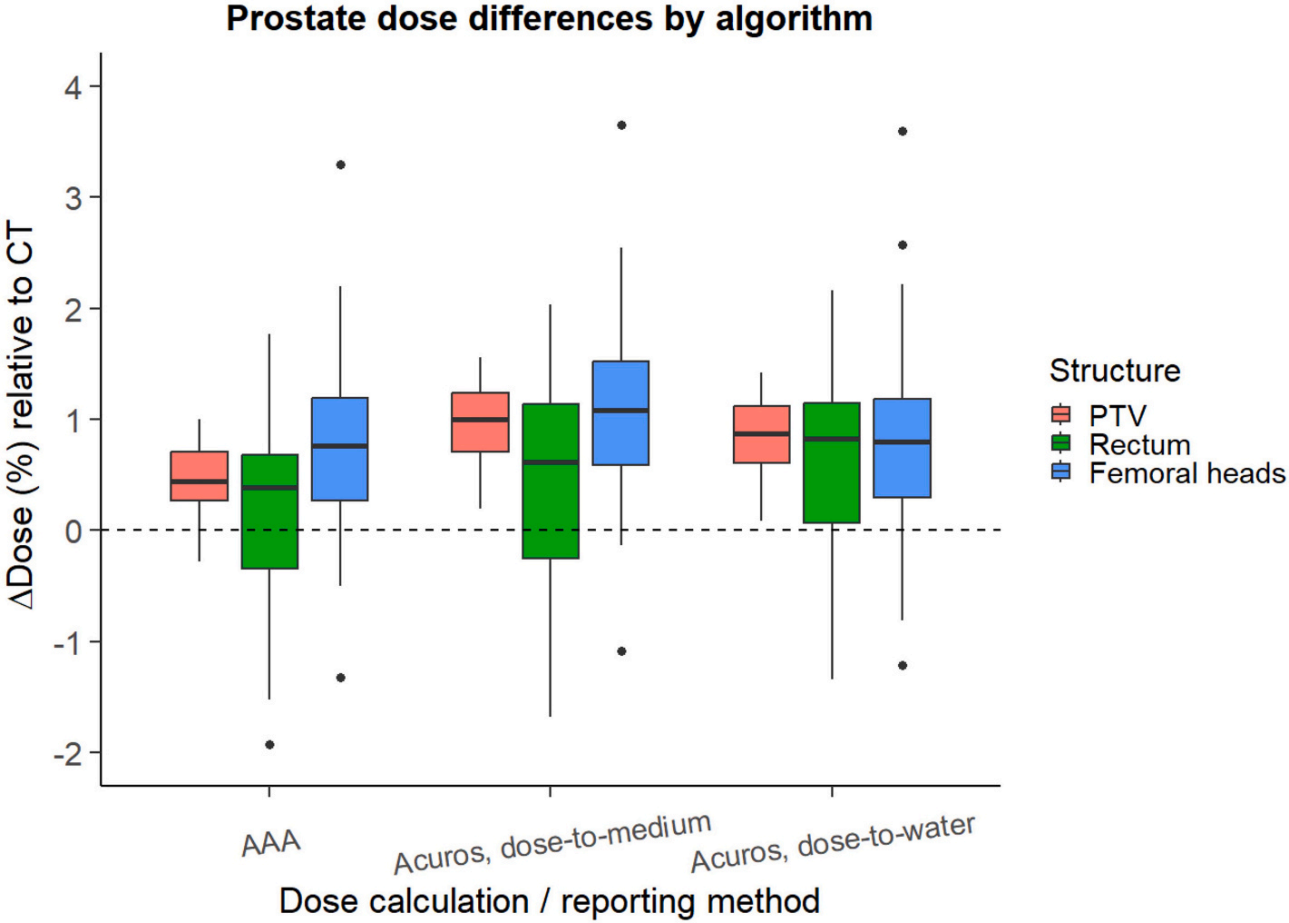
Relative dose differences (ΔDose, %) between DL-based sCT and CT for prostate patients (n = 39) calculated using AAA, Acuros XB (dose-to-medium) and Acuros XB (dose-to-water). Results are shown for the PTV, rectum, and femoral heads. For the PTV, D_95%_ was evaluated, while mean dose was used for the organs-of-interest. The dashed horizontal line visualizes zero difference relative to CT. Boxes represent the interquartile range, the central line the median, and whiskers extend to 1.5 × IQR. Points indicate outliers.

Across all evaluated structures in the prostate cohort, AAA resulted in the smallest dose differences, while Acuros XB generally showed larger deviations from CT. For example, the mean PTV D_95%_ dose difference increased from 0.5% with AAA to 1.0% with Acuros XB dose-to-medium and 0.8% with Acuros XB dose-to-water (Table 1). Furthermore, dose-to-medium produced slightly larger dose differences than dose-to-water for the target volumes and femoral heads (Fig. 1). Pairwise statistical comparisons confirmed significantly larger sCT-to-CT dose differences for Acuros XB than for AAA for all evaluated prostate target dose metrics (*p* < 0.001). The differences between Acuros XB dose-to-medium and dose-to-water were smaller, but statistically significant for several target metrics.

**Fig. 2:**
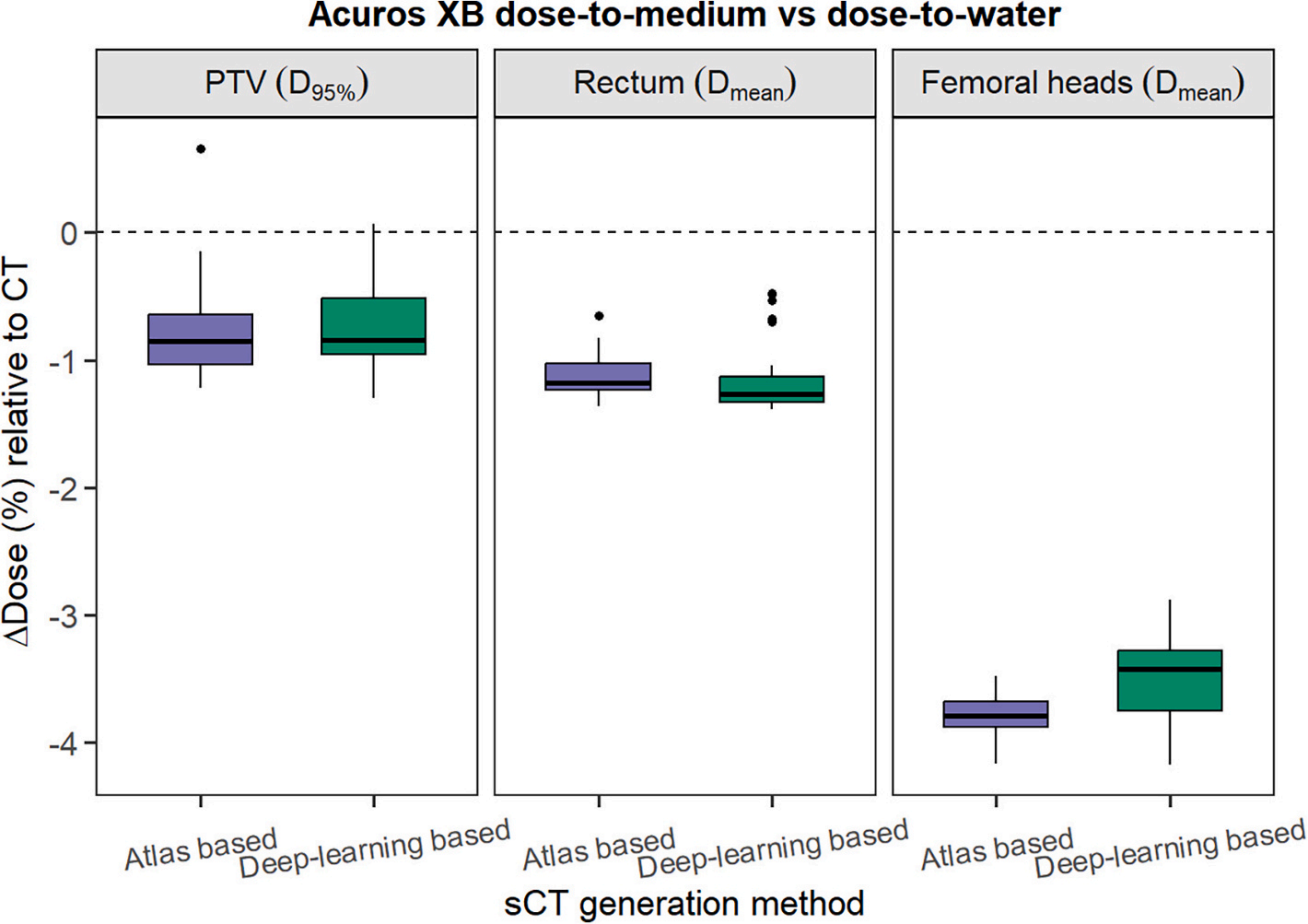
Difference between Acuros XB (dose-to-medium) and Acuros XB (dose-to-water) for atlas-based and DL-based sCTs in prostate patients (*n* = 39). Results are shown separately for the PTV, rectum, and femoral heads. D_95%_ was used for evaluating PTV and mean dose for rectum and femoral heads. The dashed horizontal line visualizes zero difference between the dose reporting methods. Boxes represent the interquartile range, the central line the median, and whiskers extend to 1.5 × IQR. Points indicate outliers.

Further analysis of the two Acuros XB dose reporting modes showed that the largest relative differences were observed in the femoral heads, with median dose differences of approximately 3.5%, whereas the corresponding differences in the rectum and PTV were approximately 1% (Fig. 2). The magnitude of the dose-to-medium versus dose-to-water difference varied only marginally between atlas- and DL-based sCTs, without a consistent pattern across the evaluated structures.

**Fig. 3:**
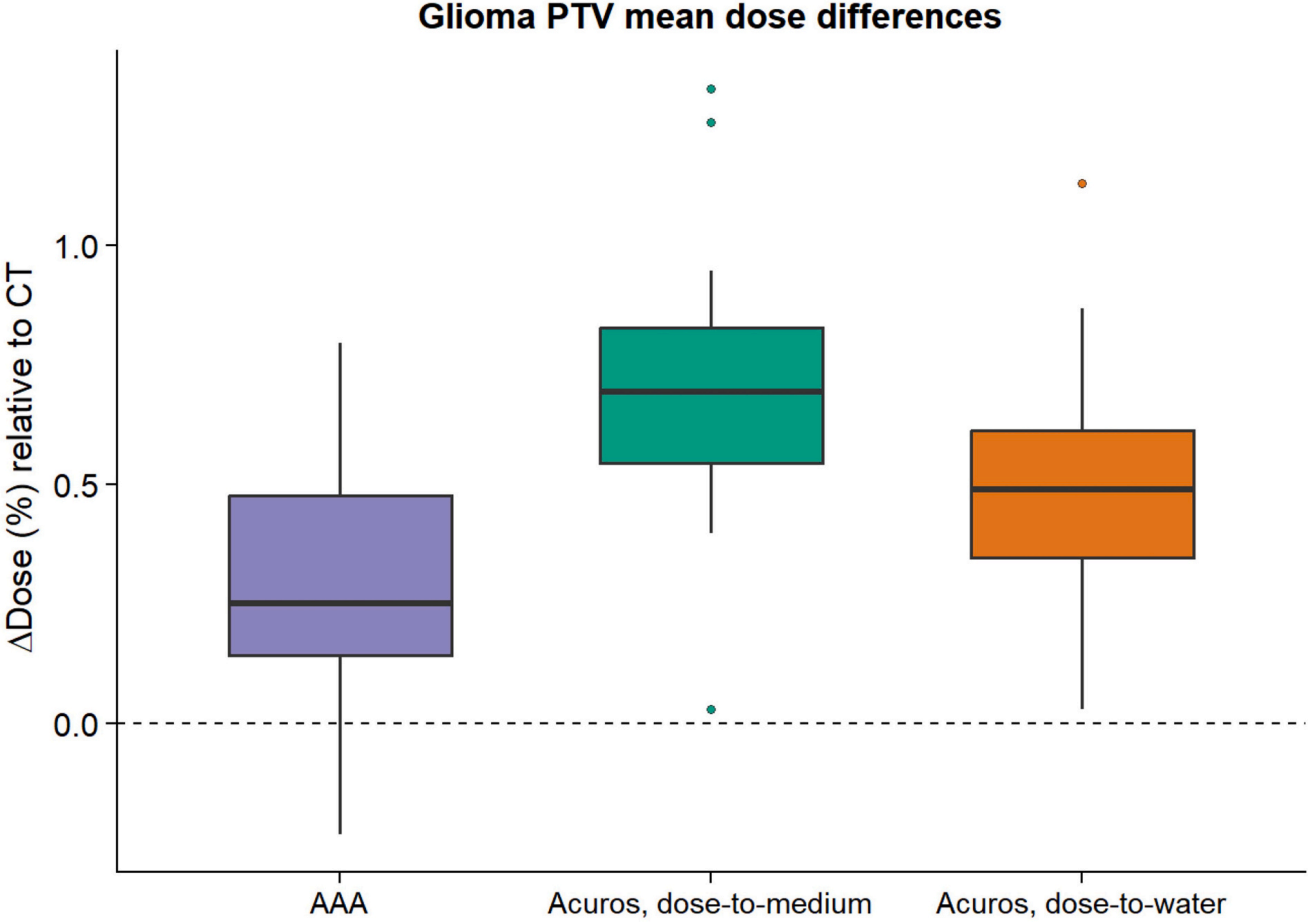
Relative PTV mean dose differences (ΔDose, %) between sCT and CT for glioma patients (n = 17). Results are presented for AAA, Acuros XB dose-to-medium, and Acuros XB dose-to-water. The dashed horizontal line visualizes zero difference relative to CT. Boxes represent the interquartile range, the central line the median, and whiskers extend to 1.5 × IQR. Points indicate outliers.

For the glioma cohort, median relative sCT-to-CT PTV mean dose differences were approximately 0.2% for AAA and 0.5% and 0.7% for Acuros XB dose-to-water and dose-to-medium, respectively (Fig. 3). Pairwise statistical comparisons confirmed significantly larger dose differences between CT and sCT for Acuros XB compared with AAA.

Comparison between the values in Table 1 and Supplementary Tab. S1 demonstrated that, in the prostate cohort, atlas-based sCT-to-CT dose differences were within 0.2 percentage points of the corresponding DL-based sCT-to-CT dose differences for both target volumes and OOIs. In both the atlas- and DL-based cohorts, the femoral heads exhibited consistently positive sCT-to-CT dose differences, with median values of approximately 1% relative to CT, compared with approximately 0.6% for the rectum, consistent with the increased sensitivity of Acuros XB (dose-to-medium) to bone density representation (Supplementary Fig. S1). Finally, the CT number override of rectal gas did not affect the majority of patients but only a few outliers (Supplementary Fig. S2).

## 4 Discussion

In this study, the dose calculation accuracy of MRI-only radiotherapy using sCT images generated with atlas- and DL-based methods for prostate and glioma patients was evaluated. The impact of dose calculation algorithm selection was specifically evaluated by comparing sCT- and CT-based dose calculations using both a convolution-superposition algorithm (AAA) and an LBTE-based algorithm (Acuros XB), the latter including both dose-to-water and dose-to-medium reporting modes. Overall, the observed dose differences between sCT- and CT-based calculations were small for both treatment sites, supporting the feasibility of MRI-only workflows when using either AAA or Acuros XB. Systematic differences were observed depending on dose calculation algorithm, dose reporting mode, and tissue type, whereas only minor differences were observed between the atlas- and DL-based sCT generation methods. The findings in this work were consistent with previously reported dose calculation errors introduced with sCTs of 0.3–2.5% [5], [20], and well below the generally accepted overall radiotherapy dose uncertainty of approximately 5% [21].

The strong statistical significance of the target dose differences between sCT and CT for both treatment sites (*p* < 0.001) is likely a reflection of the relatively low inter-patient variability and the homogeneous dose distribution within the target volumes, enabling detection of these relatively small systematic differences. AAA consistently yielded the smallest dose differences between CT and sCT, whereas Acuros XB (dose-to-medium) showed the largest, which was also confirmed by the pairwise statistical comparison between algorithms. This is consistent with the known characteristics of the algorithms: AAA, as a convolution-superposition method, has limited sensitivity to local density variations, while Acuros XB, by numerically solving the LBTE, accounts more explicitly for tissue heterogeneities [11]. This increased sensitivity is generally advantageous in anatomically heterogeneous regions, where more accurate modelling of material interfaces may improve dose calculation accuracy. The observed dose differences suggested that MRI-only workflows validated using AAA may slightly underestimate the dose deviation that may occur when using more advanced calculation methods, such as Acuros XB or Monte Carlo. This was consistent with a previous study evaluating brain MRI-only treatment [16].

For OOIs, dose differences were generally low and/or not statistically significant, indicating that the observed sCT-related CT number discrepancies had small clinical impact outside the target volumes. While the prostate cohort demonstrated minor systematic shifts, no consistent trend between sCT- and CT-based calculations was observed in the glioma cohort.

The difference between the two Acuros XB dose reporting modes has previously been shown to be tissue-dependent, with the largest relative differences observed in bone and smaller differences in soft tissue [16]. This was reflected in Fig. 2, which further indicated that DL-based sCTs tend to exhibit slightly larger dose-to-medium versus dose-to-water differences than atlas-based sCTs, consistent with an improved representation of tissue heterogeneities. However, only minor dose difference variations were observed, indicating that both sCT generation methods were suitable for MRI-only planning when used with dose calculation algorithms similar to both AAA and Acuros XB. Previous studies have also reported comparable dose calculation performance between atlas-based and DL-based sCT generation methods [22].

Several limitations in this study need to be acknowledged. First, this was a single-institution study using one commercial sCT software platform (atlas- and DL-based versions) and one treatment planning system. Secondly, the evaluation was limited to photon VMAT treatments in the male pelvic region and in the brain, not including head-and-neck or other regions with pronounced air-to-bone interfaces. Additionally, due to the rigid registration of the prostate patients and the time interval between CT and MRI acquisition, some anatomical differences between the CTs and corresponding sCTs may have influenced the dose calculation comparison. Deformable image registration was not used, as the associated interpolation and deformation uncertainties could themselves influence the observed dose calculation differences [23]. Finally, statistical significance and dose differences for the glioma cases were assessed without regard to the fact that they had different prescribed dose. However, the clinical interpretation was based primarily on the percentage difference in target dose rather than OOI dose differences and their *p*-values.

In conclusion, Acuros XB demonstrated higher sensitivity to sCT-related CT number discrepancies than AAA, with approximately two-fold larger dose differences observed in several evaluated metrics. However, despite these systematic differences between algorithms, the resulting dose deviations remained clinically acceptable for both prostate and glioma patients. These findings support the robustness of MRI-only radiotherapy, while demonstrating that the choice of dose calculation algorithm influences the magnitude of observed sCT-related dose differences.

## CRediT authorship contribution statement

**Sacha af Wetterstedt:** Writing – review & editing, Writing – original draft, Visualization, Software, Project administration, Methodology, Investigation, Formal analysis, Data curation, Conceptualization. **Emilia Persson:** Writing – review & editing, Data curation, Conceptualization. **Christian Jamtheim Gustafsson:** Writing – review & editing, Software, Methodology, Data curation. **Adalsteinn Gunnlaugsson:** Writing – review & editing, Supervision, Resources. **Minna Lerner:** Writing – review & editing, Methodology, Investigation, Data curation. **Lars E. Olsson:** Writing – review & editing, Supervision, Methodology, Conceptualization. **Tobias Pommer:** Writing – review & editing, Supervision, Methodology, Investigation, Conceptualization.

## Declaration of competing interest

The authors declare the following financial interests/personal relationships which may be considered as potential competing interests: Christian Jamtheim Gustafsson has received a speaker fee from GE Healthcare. Other authors declare that they have no competing interests.
