# Supplementary material for: Impact of dose calculation algorithm on magnetic resonance imaging-only radiotherapy for prostate and glioma
Source: Phys Imaging Radiat Oncol. 2026 Jul 20;40:101045. doi: 10.1016/j.phro.2026.101045 (PMC13473937; doi:10.1016/j.phro.2026.101045)
Supplement: Supplementary file 1 [file mmc1.pdf]

Supplementary material

Impact of dose calculation algorithm on magnetic resonance imaging-only radiotherapy for prostate and glioma

Table S1. *Mean dose differences between sCT- and CT-based dose calculations for prostate patients (n = 39, prescribed dose 78 Gy/39 fr) along with ranges within brackets for clinically used dose metrics. Target volumes (PTV and CTV) are expressed as relative percentage differences, while organs-of-interest dose criteria are reported as absolute differences in Gy or percentage points (pp). L=left, R=right. Dose calculations were performed using Acuros XB (dose reporting: both dose-to-medium and dose-to-water) and AAA. P-values were derived from paired t-tests.*

Mean dose difference between atlas-based sCT and CT – Prostate

	**AAA**	**p-value**	**Acuros XB, dose-to-medium**	**p-value**	**Acuros XB, dose-to-water**	**p-value**
PTV $D_{95\%}$ (%)	0.4 (-0.3 to 1.1)	<0.001	1.0 (0.2 to 1.7)	<0.001	0.9 (-0.1 to 1.6)	<0.001
PTV D_{mean} (%)	0.4 (-0.3 to 1.1)	<0.001	0.9 (0.2 to 1.6)	<0.001	0.8 (-0.5 to 1.4)	<0.001
CTV $D_{99\%}$ (%)	0.5 (-0.2 to 1.2)	<0.001	1.4 (0.6 to 2.4)	<0.001	0.9 (-1.2 to 1.7)	<0.001
Rectum V_{70Gy} (pp)	0.2 (-0.7 to 0.7)	0.003	0.3 (-0.5 to 1.0)	<0.001	0.4 (-0.3 to 1.0)	<0.001
Bladder V_{65Gy} (pp)	0.2 (-1.2 to 1.0)	<0.001	0.3 (-1.1 to 1.1)	<0.001	0.4 (-1.1 to 1.6)	<0.001
Bladder D_{mean} (Gy)	0.2 (-1.7 to 1.0)	0.007	0.3 (-1.7 to 1.0)	<0.001	0.3 (-1.7 to 1.1)	<0.001
Femoral head L $D_{2\%}$ (Gy)	0.1 (-0.3 to 0.6)	<0.001	0.3 (-0.3 to 0.9)	<0.001	0.2 (-0.2 to 0.6)	<0.001
Femoral head R $D_{2\%}$ (Gy)	0.1 (-0.2 to 0.6)	<0.001	0.2 (-0.1 to 0.7)	<0.001	0.1 (-0.3 to 0.7)	0.004
Femoral head L D_{mean} (Gy)	0.1 (-0.3 to 0.4)	<0.001	0.2 (-0.2 to 0.5)	<0.001	0.1 (-0.3 to 0.5)	<0.001
Femoral head R D_{mean} (Gy)	0.1 (-0.2 to 0.4)	<0.001	0.2 (-0.1 to 0.5)	<0.001	0.2 (-0.1 to 0.5)	<0.001

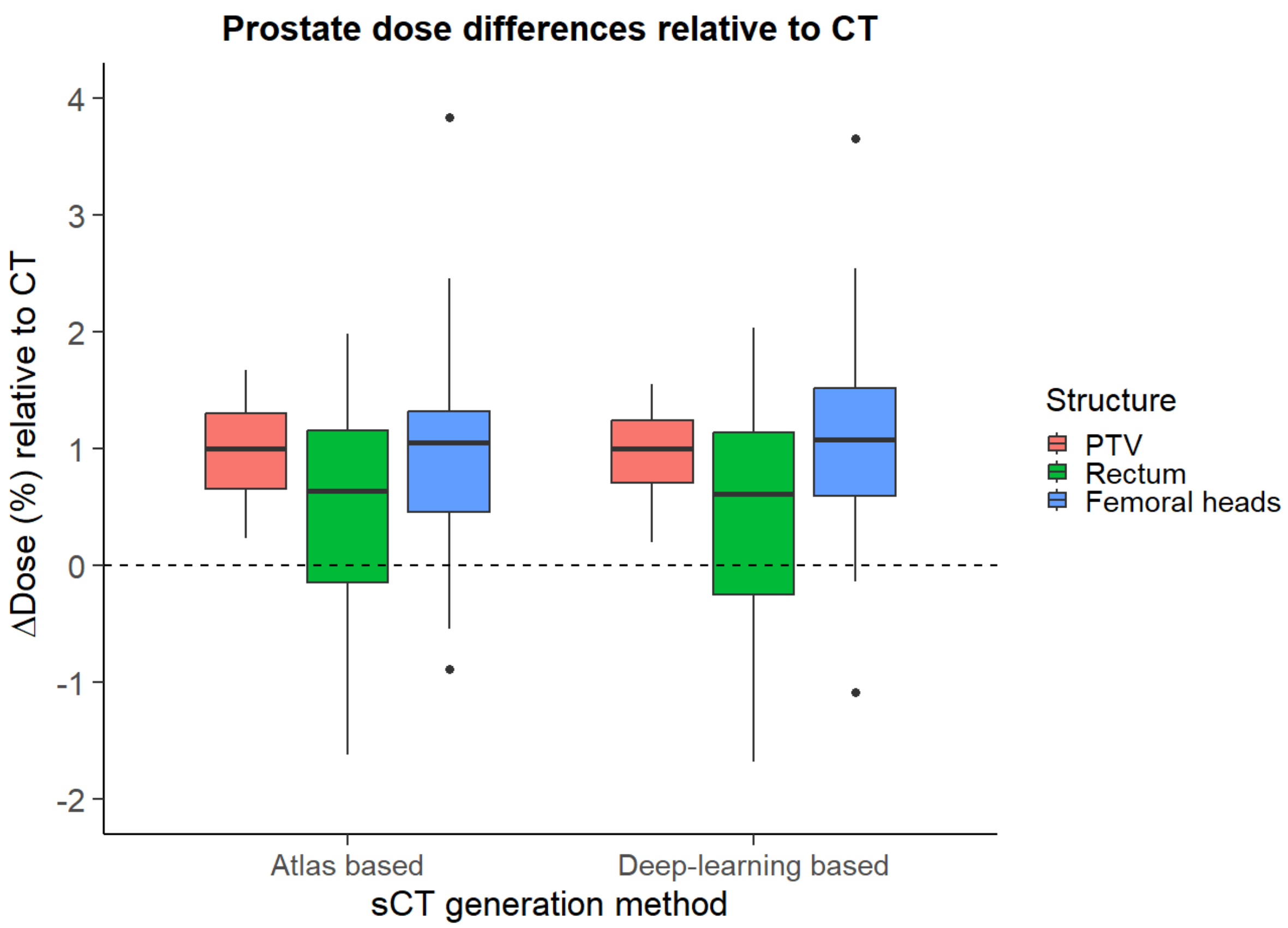

Figure S1. *Boxplots showing the relative dose difference (ΔDose, %) between synthetic CT and conventional CT for prostate patients (n = 39), calculated with Acuros XB (dose-to-medium). Results are shown for target volume (PTV), rectum, and femoral heads. Atlas-based and DL-based sCT generation methods are compared in the figure. For PTV, $D_{95\%}$ was evaluated, while mean dose was used for organs-of-interest. The dashed horizontal line visualizes zero difference relative to CT. Boxes represent the interquartile range (IQR), the central line the median, and whiskers extend to 1.5×IQR. Points indicate outliers.*

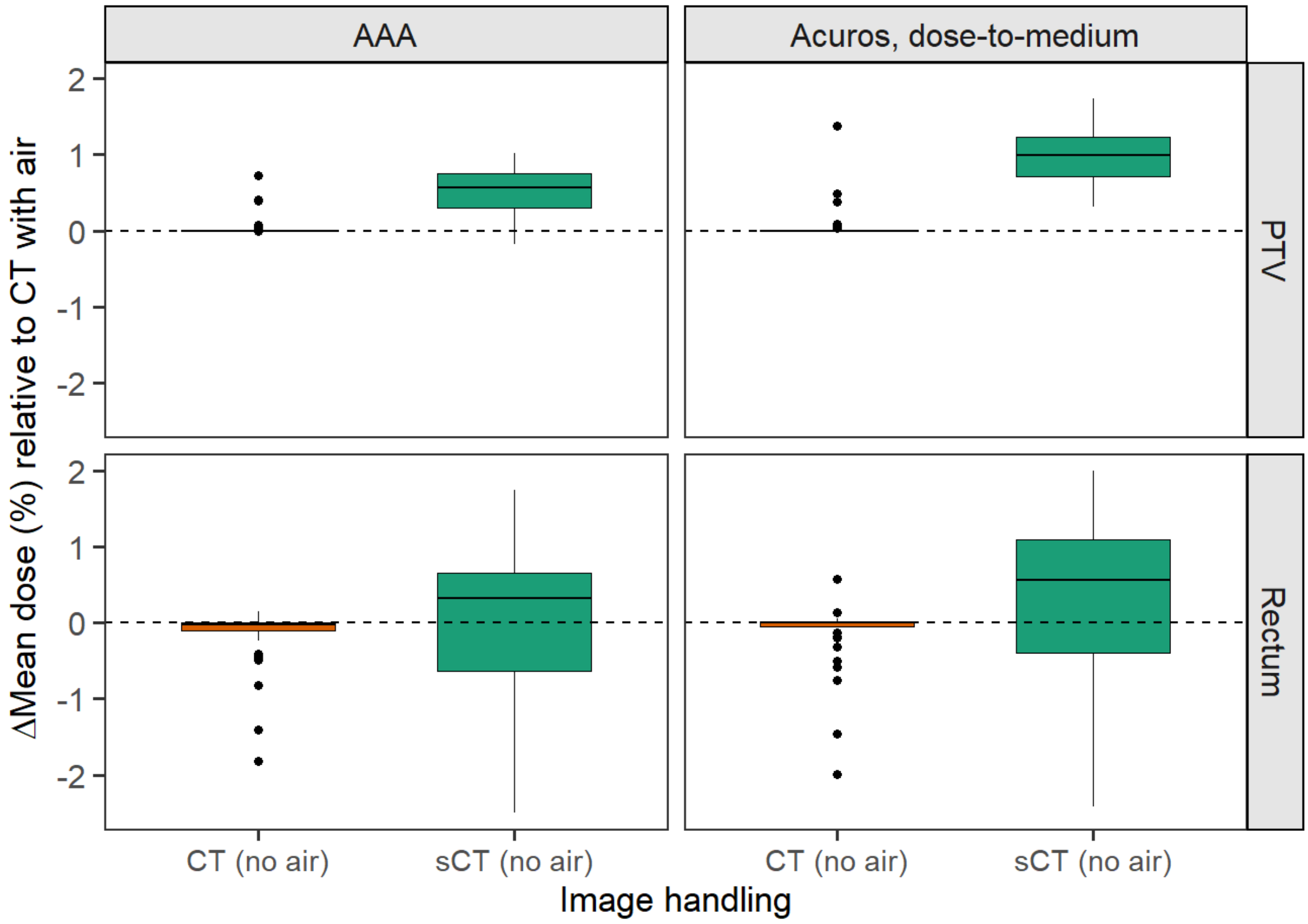

Figure S2. *Boxplots showing the relative mean dose difference (ΔDose, %) compared to CT with rectal air for prostate patients, evaluating the impact of rectal air on dose calculation. Results are presented for PTV and rectum using AAA and Acuros XB (dose-to-medium). For each algorithm, there are two comparisons: CT with rectal air overridden to water ("CT (no air)") and DL-based sCT, which inherently is without air, ("sCT (no air)"), both calculated relative to the original CT with air. The dashed horizontal line indicates zero difference relative to CT with air. Boxes represent the interquartile range, the central line the median, and whiskers extend to 1.5×IQR. Points indicate outliers.*
